# Automatic rape flower cluster counting method based on low-cost labelling and UAV-RGB images

**DOI:** 10.1186/s13007-023-01017-x

**Published:** 2023-04-24

**Authors:** Jie Li, Enguo Wang, Jiangwei Qiao, Yi Li, Li Li, Jian Yao, Guisheng Liao

**Affiliations:** 1 Hubei Key Laboratory for High-efficiency Utilization of Solar Energy and Operation Control of Energy Storage System, Hubei University of Technology, 430068 Wuhan, China; 2 Oil Crops Research Institute of the Chinese Academy of Agricultural Sciences, Key Laboratory of Biology and Genetic Improvement of Oil Crops, Chinese Academy of Agricultural Sciences, Wuhan, China; 3 School of Remote Sensing and Information Engineering, Wuhan University, Wuhan, China; 4 National Lab of Radar Signal Processing, Xidian University, Xian, China

**Keywords:** Rape flower clusters, Pyramidal convolution, Attention mechanism, Bayesian loss

## Abstract

**Background:**

The flowering period is a critical time for the growth of rape plants. Counting rape flower clusters can help farmers to predict the yield information of the corresponding rape fields. However, counting in-field is a time-consuming and labor-intensive task. To address this, we explored a deep learning counting method based on unmanned aircraft vehicle (UAV). The proposed method developed the in-field counting of rape flower clusters as a density estimation problem. It is different from the object detection method of counting the bounding boxes. The crucial step of the density map estimation using deep learning is to train a deep neural network that maps from an input image to the corresponding annotated density map.

**Results:**

We explored a rape flower cluster counting network series: RapeNet and RapeNet+. A rectangular box labeling-based rape flower clusters dataset (RFRB) and a centroid labeling-based rape flower clusters dataset (RFCP) were used for network model training. To verify the performance of RapeNet series, the paper compares the counting result with the real values of manual annotation. The average accuracy (Acc), relative root mean square error (rrMSE) and $R^2$ of the metrics are up to 0.9062, 12.03 and 0.9635 on the dataset RFRB, and 0.9538, 5.61 and 0.9826 on the dataset RFCP, respectively. The resolution has little influence for the proposed model. In addition, the visualization results have some interpretability.

**Conclusions:**

Extensive experimental results demonstrate that the RapeNet series outperforms other state-of-the-art counting approaches. The proposed method provides an important technical support for the crop counting statistics of rape flower clusters in field.

## Background

Rapeseed oil is the third largest vegetable oil in the world and one of the main sources of vegetable oil for human consumption [1]. Prospective observational studies have demonstrated that vegetable oils are protective against cardiovascular disease (CVD) and that canola oil has the potential to improve many cardiometabolic risk factors [2]. The global rapeseed supply is expected to grow by $10\%$ between 2022 and 2023, with crushing volumes reaching a record 75.1 million tons [3]. Rapeseed is the most productive oilseed crop in China [4]. However, constrained by natural resources and the rapid progress of urbanization, China’s cultivated land area continues to decrease. The sown area of rapeseed decreased from 7.192 million hectares in 2011 to 6.8 million hectares in 2020 [5]. Consequently, continuously increasing rapeseed production plays an important role in ensuring the supply of rapeseed oil.

Flowering stage is crucial to its growth. For example, chemical pest control on newly flowering rape plants can effectively manage rape flower beetles and other pests to guarantee optimum rape plant growth and flowering [6]. Foliar spraying of plants at different flowering periods to provide the critical nutrients required for blossoming oilseed rape is a crucial part of enhancing the yield and quality of oilseed rape [7, 8]. The flowering of oilseed rape help farmers manage their fields better. In addition, the number of pods of rapessed per plant is decisive for seed yield. This trait is ultimately determined by the survival of flowers [9]. The quantization for flower clusters is imperative in precision agriculture, which can help to predict the yield information of oilseed rape in the corresponding fields for agriculturist and breeder [10, 11]. However, field rape flower cluster counting relies on manual counts, which are labor- and time-intensive. The rape flower count results are subordinate to subjective bias, making it more challenging to monitor rape growth in a large field scenarios [12–14]. Precision agriculture needs advanced field technology. For this reason, it is crucial to further extend the study of an automatic and non-destructive technology to count the number of rape flower clusters.

Previous studies have been paying close attention to use of remote sensing technology in high-throughput flower phenotypic analysis [15–17]. Fang et al. [18] captured canopy reflections in green, red, and red-edged, NIR bands of rape by a multispectral system mounted on an unmanned aerial vehicle (UAV). This work achieved the estimation of rape vegetation and rape flowers. Wan et al. [19] combined vegetation indices (VIs) extracted from RGB and multispectral images and image classification to estimating flower number in oilseed rape. Zang et al. [20] developed an enhanced area yellowness index (EAYI) based on Moderate Resolution Imaging Spectroradiometer (MODIS) time series data for mapping rape flowers. Zhang et al. [21] investigated the application of vegetation indices in estimating canola flower numbers. However, few studies focus on the quantity acquisition of flowers directly from the image using remote sensing technology. Spatial resolution is a challenge for multispectral and satellite imaging, especially when counting small objects.

Oilseed rape is a crop species with remarkable flowers during growth [18]. Sulik et al. [22] reported that the band ratio of green and blue light was strongly (r^2^ = 0.87) related to the number of yellow flowers per unit area. Consequently, the UAV with RGB imaging characterized by high resolution and flexible acquisition is an effective way to count flower clusters. Deep learning methods for crop counting in RGB imaging have been presented in recent years [23, 24]. Samiei et al. [25] designed a deep learning CNN network to learn the cotyledon opening during plant seedling development. Jiang et al. [26] used the Faster-RCNN model with the Inception ResNet v2 feature extractor, which can accurately calculate field plant seedlings. Yang et al. [27] introduced a Yolov4-based spatial pyramid pooling (SPP) and multi-level feature fusion method with substantial improvement in counting performance. The method mentioned above uses the detected object boxes to count. Outputs are the locations of individual instances and their corresponding bounding boxes. Nevertheless, when it comes to counting the flowering of rape, the number of flower clusters is anywhere from a few to a thousand in one plot. Especially at peak flowering, objects are dense and overlapping. They were difficult to detect clearly. Additionally, dense object has caused great trouble for the labelling work when using the deep learning method.

Recently, a deep neural network called TasselNet [28] used a regression counting approach that objects in an image were described by a density map given dot annotations performed well on maize counts. After that, the TasselNetv2 [29], TasselNetV2+ [30], and TasselNetV3 [31] networks were proposed to further improve the counting performance by redesigning the normalizer and introducing image segmentation sub-networks. These counting methods have yielded good results on crop datasets such as the wheat ear dataset [32] and rice planting dataset [33]. In particular, for images of large crowds, this density map estimation approach has been shown to be more robust than the detection-then-counting approach [34]. The shape of rape flower clusters is nearly round, which makes them more suitable for dotted labelling and counting. But there has not been a study on the rape flower cluster counting using a regression approach.

The main motivation of this study is to develop an automatic counting method using deep learning method with low-cost labelling based on UAV-RGB images. The objective is to: (I) build and train a lightweight deep regression network for rape flower cluster counting, (II) construct the homemade rape flower cluster dataset for training the proposed model, (III) evaluate the performance of different counting method with manual counting, (IV) verify the effectiveness of the proposed method in field.

## Materials and methods

**Fig. 1:**
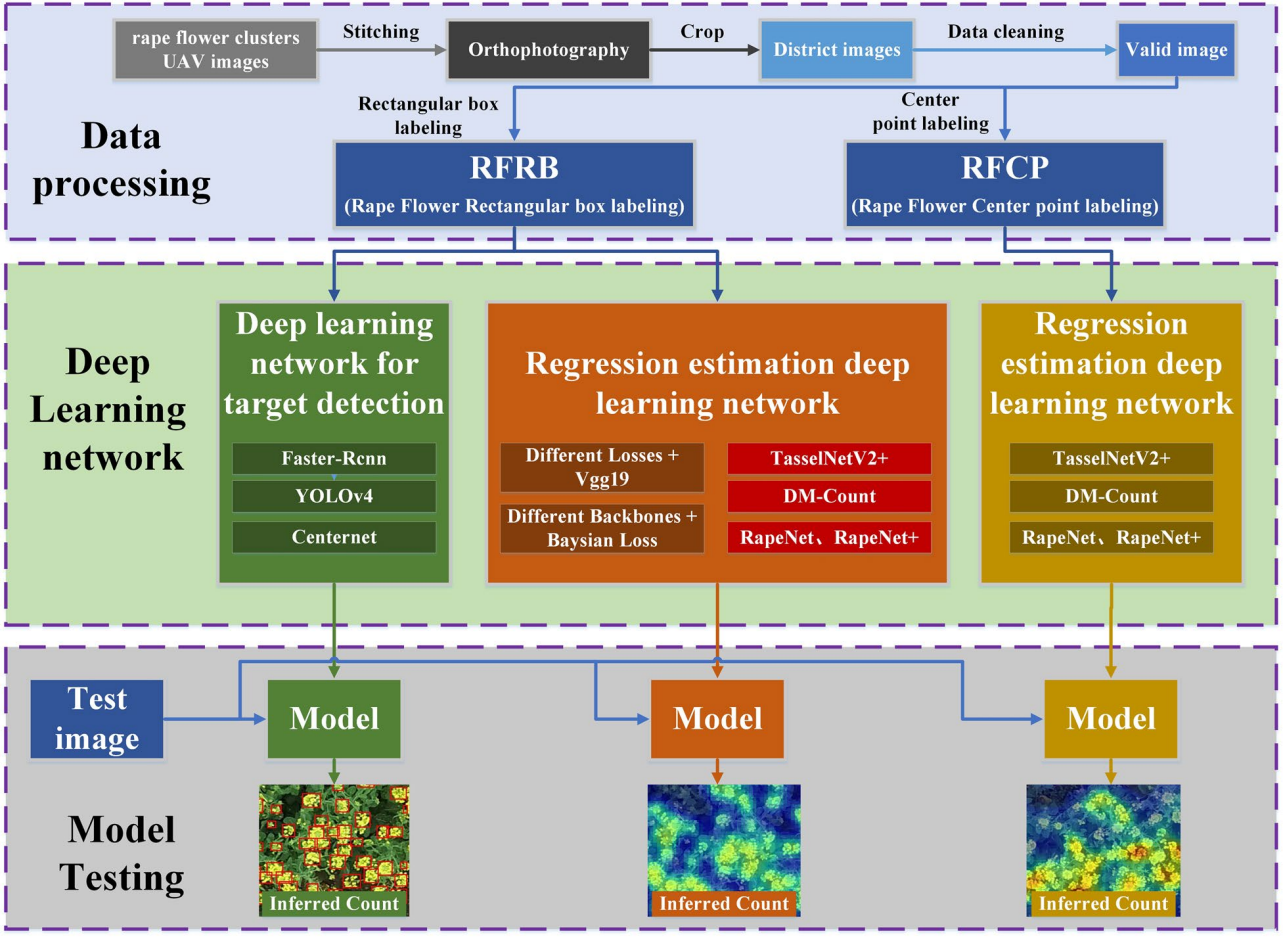
Schematic diagram of applying deep learning network for UAV rape flower clusters counting

This paper explores the application of the deep learning method in rape flower cluster counting, which is divided into three main parts: data processing, deep learning network building, and model testing, as detailed in Fig. 1.

In the data processing part, we stitched the rape cluster images taken by the UAV to obtain the orthophotos. The orthophotos were cropped by planting plot to obtain rapeseed cluster images for each small plot. The rape cluster images of small plots were then used for data cleaning to obtain a valid rape cluster image. We use rectangular box annotation and centroid annotation to obtain two canola cluster annotation datasets, RFRB and RFCP, respectively.

In the section on deep learning network construction, we studied two types of counting methods: object detection and regression estimation. On the RFRB dataset, we conducted a rape flower cluster counting study on these two types of methods. Only regression estimation deep learning networks are used on the RFRP dataset.

For the model testing part, we compared the evaluation metrics coming from object detection and regression estimation and performed a correlation analysis. To further analyze the reliability of the counts, we visualized the output of the two types of networks in the form of bounding boxes and heat maps, respectively, based on the output count results.

### Study area and image acquisition

**Fig. 2:**
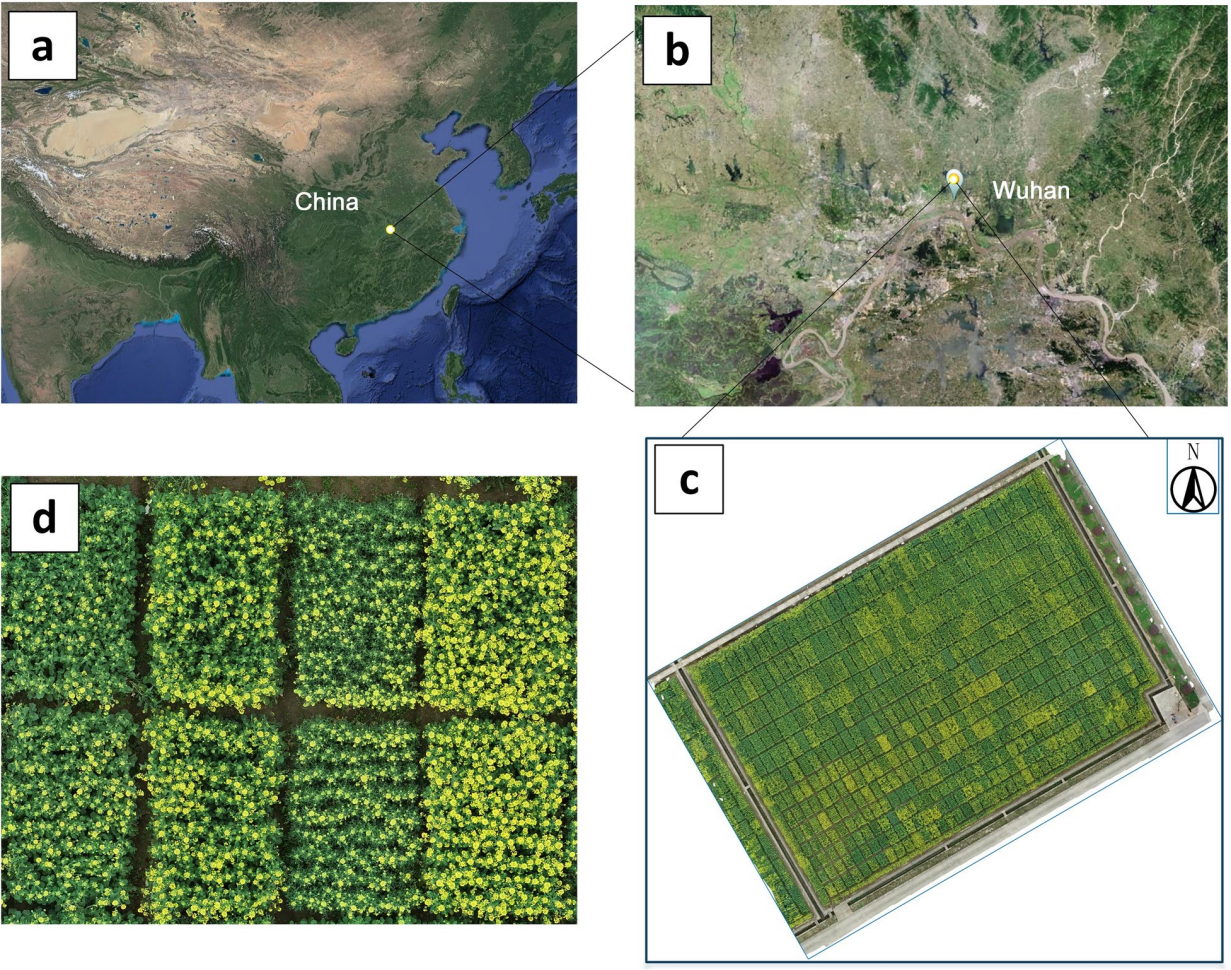
Geographical location and UAV remote sensing image mosaic map of the study area. **a** Location of the study area in Asia. **b** Location of the experimental area in Wuhan City. **c** Stitching map of UAV images in the experimental area. **d** Enlarged images of oilseed rape images in some plots

Winter rape (seeded at the end of September and harvested in May) and spring rape are the two types of oilseed rape (planted at the end of April and harvested in September). In terms of planting area and productivity, winter rape occupies more than 90% of China and 1/4 of the world, with the majority of it being grown in the Yangtze River basin. The experimental area was located at the Yangluo base of the Institute of Oilseeds, Chinese Academy of Agricultural Sciences, Xinzhou District, Wuhan City, Hubei Province, China (N$30^\circ 71^\prime$, E $114^\circ 51^\prime$) (Fig. 2). Wuhan is located in the eastern part of Jianghan Plain and the middle reaches of Yangtze River. Its climate type is subtropical monsoon (humid) climate at an altitude of about 24 m, with annual precipitation of 1150–1450 mm and annual average temperature of 15.8–-$17.5^{\circ }\hbox {C}$. These provide a suitable growth environment for rape planting.

The experimental field was divided into 252 plots, and the plot area was divided into 8 m$^2$ (2 m × 4 m) and 6 m$^2$ (2 m × 3 m). Oilseed rape in these plots was managed in the same field management mode with regular irrigation and weeding. The oilseed rape used in the experiment was winter rape, and the UAV shooting period was from February 2021 to May 2021, when rape photosynthesis is strong.

**Fig. 3:**
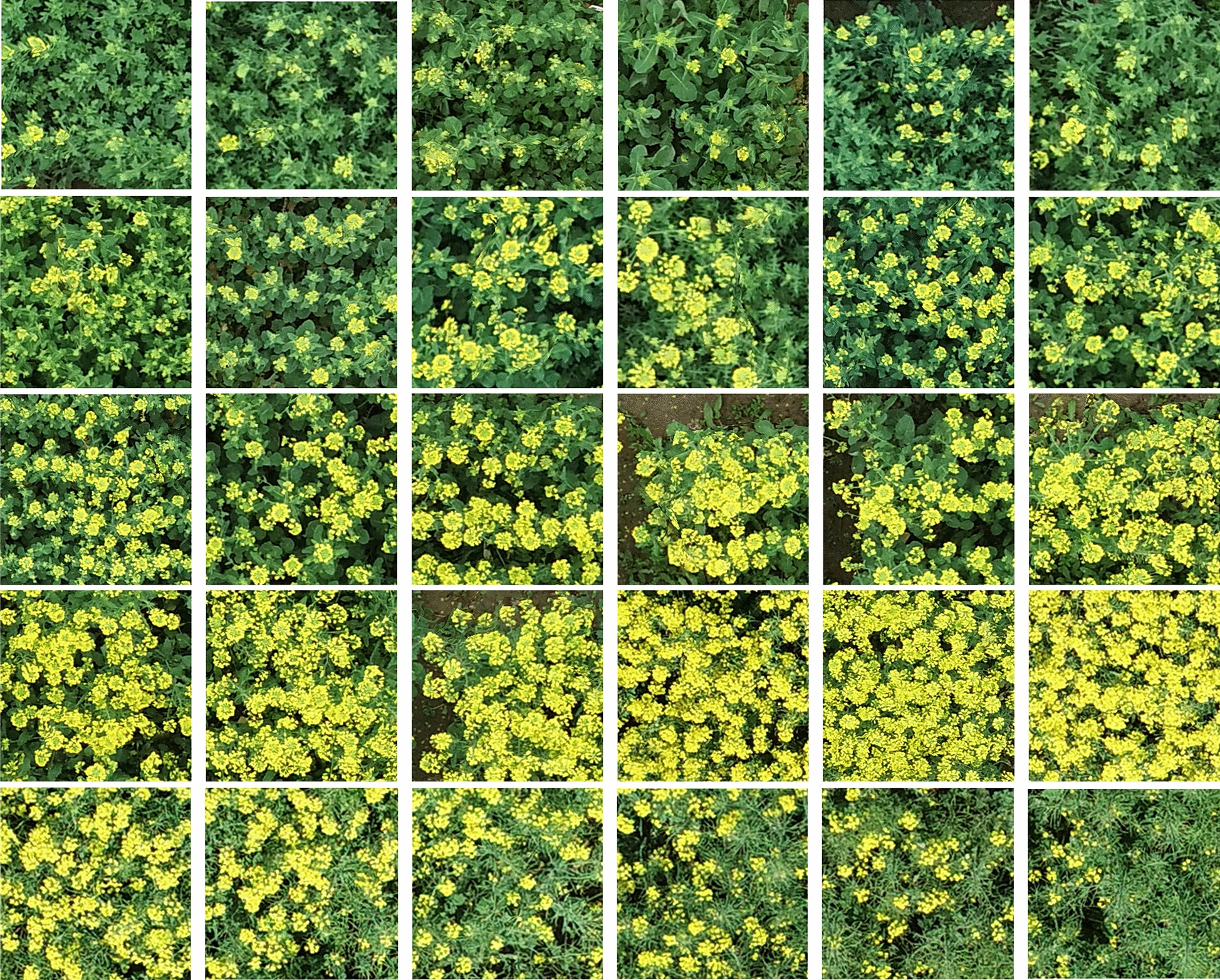
Geographical location and UAV remote sensing image mosaic map of the study area. From the top left to the bottom right are sample images of rape flower clusters taken during the bud, early flowering, full flowering and decaying stages

In order to sufficiently obtain the morphological characteristics of rape flowers at different periods, the UAV took image data of rape at different flowering periods. Images of rape flower clusters at bud stage, first flowering stage, full flowering stage and decaying stage are shown in Fig. 3, respectively. From the image, we can see that as rape gradually enters the peak flowering period, the flower clusters become denser and difficult to distinguish.

**Table 1:** UAV image collection environment

Year	Acquisition dates	Growths	Flight altitude (m)	Temperature ($^{\circ }\hbox {C}$)	Environment
2021	February 19	Bud stage	15	12	11–13 *am*; low/middle cloudy or cloudless; wind speed less than 4*m*/*s*; Good light conditions
	February 26	First flowering stage	15	14	
	March 3	Full flowering stage	10	21	
	March 14	Full flowering stage	10	23	
	March 22	Decaying stage	10	24	
	April 5	Decaying stage	10	15	
2022	March 1	Full flowering stage	13	20	

The UAV model used for data acquisition was Phantom 4pro V2.0, the camera is 2$\times$ $10^7$ pixels, and the size of the single image was 5472$\times$3648. Image acquisition of each plot was carried out under natural conditions by remotely operated UAVs carrying RGB cameras. Automatic planning and aerial photography mode were adopted. The course overlap rate and side overlap rate were set to $75\%$. The flight altitude was set to a fixed value each time and the flight speed was set to 1.9 m/s. Data collection in the study area was completed in about 100 min. In order to ensure that the captured images met the experimental criteria, we screened the initially acquired images and found that the quality of the rape images taken in the afternoon, evening, or when the clouds were heavily obscured was poor due to the influence of incident light. The data acquisition environment is shown in Table 1. We chose to shoot between 11 and 13 a.m., considering the optimal lighting conditions and wind speed of less than 4 m/s.

### Rape-flower-cluster datasets preparation

We opt for a supervised learning strategy for the rape flower clusters counting study in an effort to get better counting results. As a result, the rape flower cluster datasets have been created and will be utilized for training. Since images acquired by UAV cannot be directly used for annotation, pre-processing operations have to be performed. The original images with a resolution of 5472$\times$ 3648 taken by the UAV are stitched together according to the geographical location of the site to form a field image with a resolution of 40485 $\times$ 27129. Then, the orthophoto is cropped according to the actual ground size using image processing software to obtain a plot image with a resolution of 606 $\times$ 1105$\sim$672 $\times$ 1266. Convert the cropped image from RGB color space to HSV color space. Increase or decrease the brightness and contrast of each image by 10$\%$ to increase the diversity of the data. This was done in order to take into account the various effects that various weather conditions have on image brightness during the data acquisition process.

**Fig. 4:**
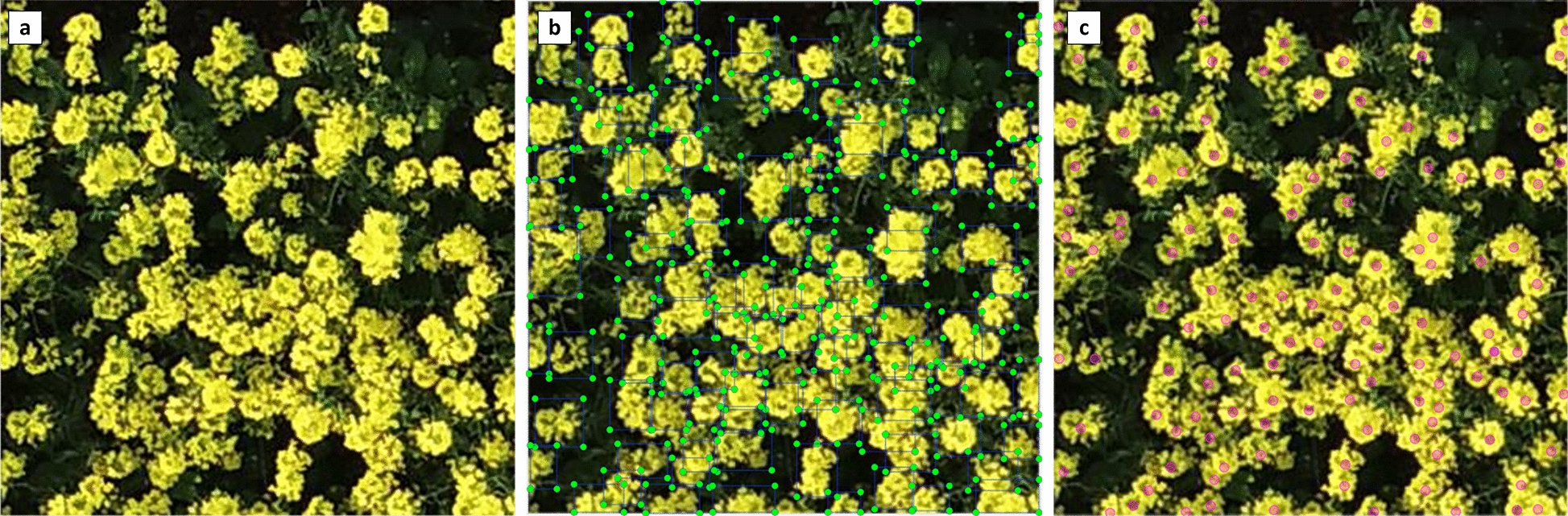
Two methods of labeling rape flower clusters. **a** Original image. **b** Rectangular box labeling of a rape flower clusters. **c** Center point labeling of a rape flower clusters

Two annotation techniques, rectangular box annotation and centroid annotation, are used in this study to produce training datasets in order to test the counting performance of different deep learning approaches. The rectangular box annotation uses the free open source annotation tool LABELIMG (https://github.com/heartexlabs/labelImg), and the center point annotation uses the free open source annotation tool LABELBEE (https://github.com/open-mmlab/labelbee-client), both of which allow simultaneous access by different users and can be used by all institutions. The original unlabeled rape flower cluster images contain clusters of complex scale and diverse traits (Fig. 4a). The rectangular box annotation requires the annotator to carefully grasp the border of the rape flower clusters (Fig. 4b), which is more difficult to operate and prone to omission due to the serious overlap and adhesion when the rape flower clusters are dense. In contrast, the center point annotation only requires the annotator to judge the center point of the rape flower clusters (Fig. 4c), which is less difficult and faster.

We obtained the rectangular box labeling dataset RFRB (Rape Flower Rectangular Box Labeling) and the center point labeling dataset RFCP (Rape Flower Center Point Labeling) by manual labeling. The minimum and maximum rape flower clusters in dataset RFRB were 8 and 686, with a median and mean of 297 and 310, respectively. The lowest and highest rape flower clusters in RFCP (Rape Flower Center point labeling) were 303 and 1198, with a median and mean value of 566 and 607, respectively.

### RapeNet

In the field of computer vision recognition, convolutional neural networks have shown superior performance in many popular areas by virtue of their high efficiency in feature extraction. Convolutional neural networks have undergone significant development over the past half-century, progressing from the earliest LeNet [35] through AlexNet [36], VGG(16)Net [37], GoogleNet [38], Microsoft ResNet [39], and so forth. The performance of network detection is positively impacted by the stacking of more convolutional layers, the use of convolutional kernels of various sizes, and the addition of multi-level residual structures. On numerous well-known large-scale datasets, existing deep convolutional neural networks have produced excellent results. However, these networks also have a number of drawbacks that are very inconvenient for practical applications, including a large model size and slow operation caused by the complexity of the network structure.

To better address the aforementioned challenges, we designed a network structure using the regression for rape flower clusters counting, which is referred to as RapeNet. The input to RapeNet is a 512$\times$1024 pixel RGB image of rape flower clusters, and the output is the counting result of the test rape flower image as well as a heat map. The pseudo-algorithm of RapeNet is given in Table 2.

**Table 2:** The pseudo-algorithm of RapeNet

Algorithm 1 RapeNet	
**Input**: A UAV-RGB image of one plot in a field.	
**Output**: The number of rape flower clusters in the image and a heat map.	
**Phase 1**: The input image $K_{i}$ is adjusted to a resolution of 512$\times$1024 image $K_{r}$, set the sliding window resolution 256$\times$256, and get 8 sub-images.	
**Phase 2**: The whole backbone network is built by pyramidal convolution. A Bayesian loss function is used to constrain the entire training process. A likelihood function is constructed for each annotated point using a Gaussian.	
**Phase 3**: Train and test the RapeNet network model.	

In Table 2, RapeNet consists of two main parts: the backbone network and the loss function. The backbone network is built using six pyramidal convolution blocks, and the loss function uses Bayesian loss. Each module is explained in detail in the following.

#### Pyramidal convolution

**Fig. 5:**
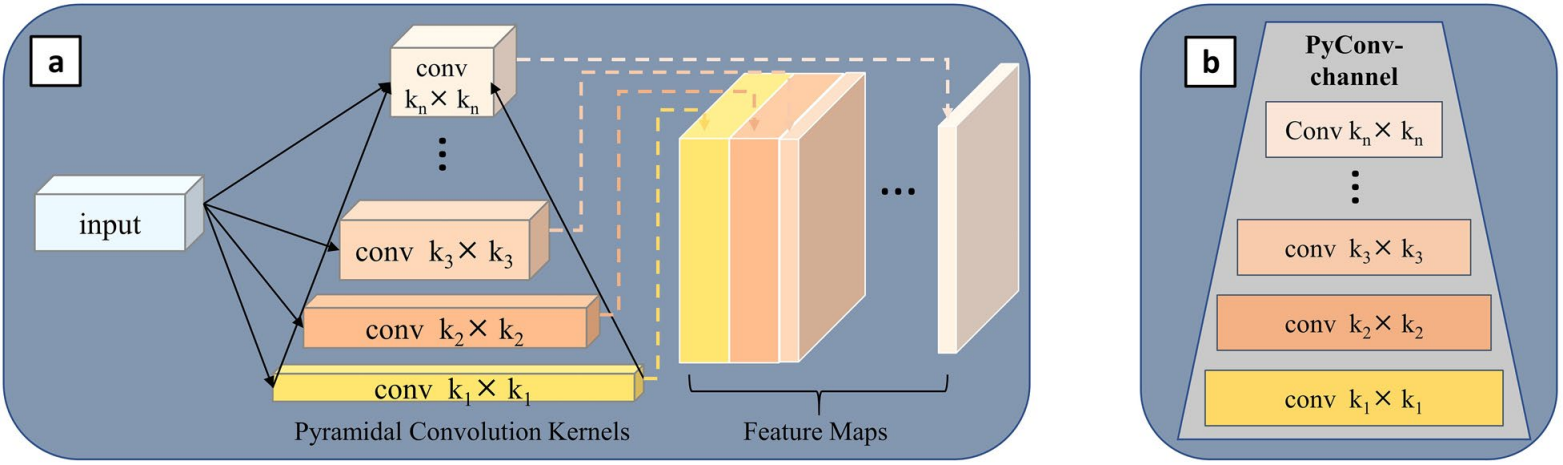
Pyramidal convolution blocks

The core of CNNs is convolution, which determines the level of feature extraction. Most CNNs use small convolutional kernels, because increasing the convolutional kernels would bring a huge cost in terms of the number of parameters and computational complexity. Ionut et al. [40] proposes a pyramidal convolutional layer to solve this problem. The kernel pyramidal structure allows for the good extraction of detailed features at different levels in the scene, which greatly improves the performance of the network without increasing the computational cost. Inspired by this, RapeNet uses the pyramidal convolutional network’s structure, which is depicted in Fig. 5a. Each level of the PyConv $\{1,2,3, \ldots , n\}$ applies different kernels with a different spatial size for each level. After passing through various convolutional kernels, the input features are stitched together to produce the final output features, which are depicted in a simplified manner in the later network structure on the right, as is shown in Fig. 5b.

**Fig. 6:**
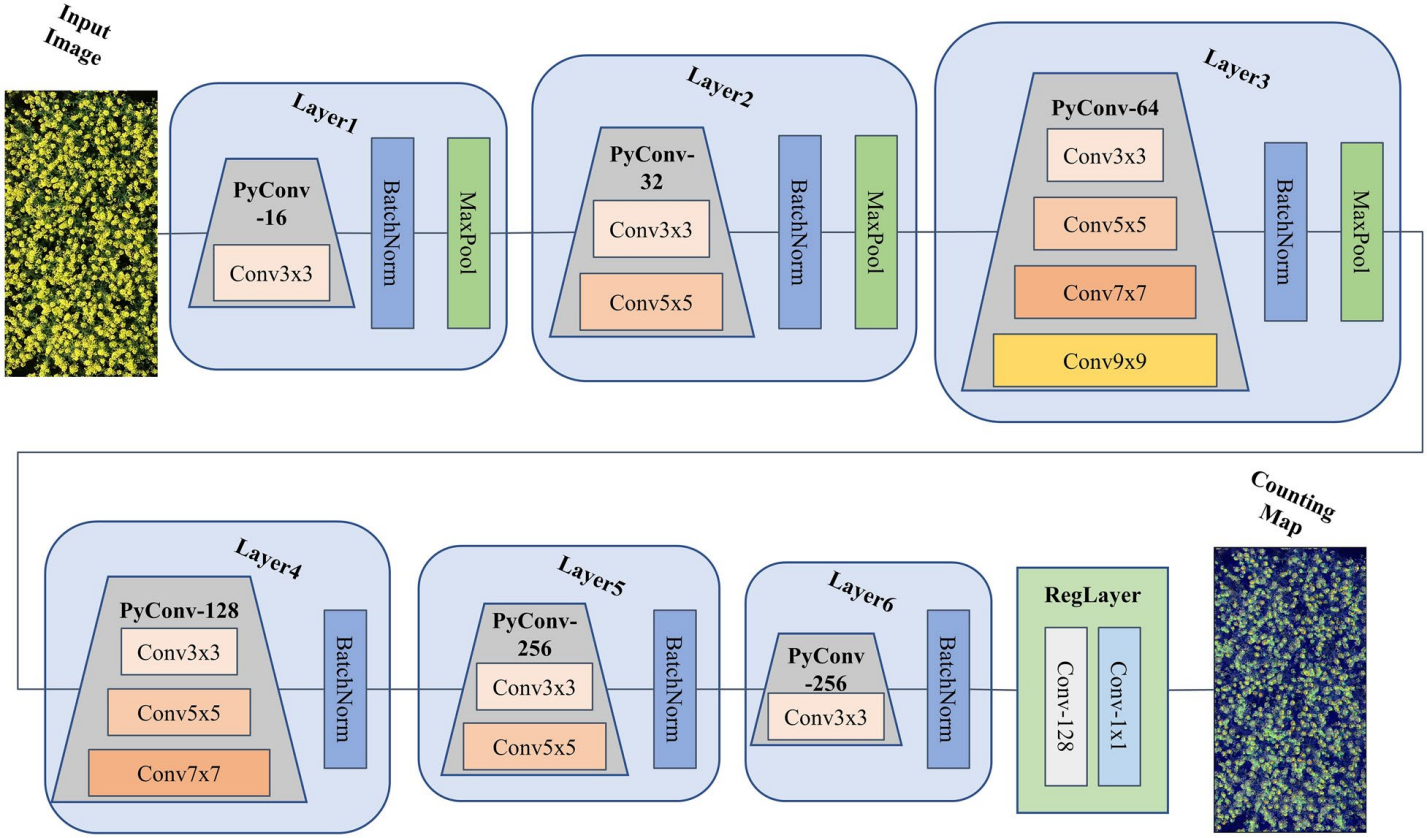
RapeNet network structure

The RapeNet network is built with six-layer pyramidal convolutional blocks to better extract detailed features and multi-scale information in each feature channel. The number of pyramidal convolutional kernels is increased in the first three blocks, then decreased in the next three blocks. Finally, the regression module is used to get the output count heat map, as shown in the Fig. 6.

#### Bayesian loss

The annotation points in our created rape flower datasets are in the center of the flowers, and the advanced approach is to turn the annotation points into density maps for regression estimation using Gaussian kernels. Manual labeling tends to have a small number of errors with the trait variation, shading, and adhesion of rape flowers. The density map converted from labeled points by Gaussian kernels is not of high quality, resulting in larger biases in regression counts on the density map. Ma et al. [41] proposed a bayesian loss function to constrain the regression training process and used labeled points to construct a density contribution probability model. Then the expected count at each annotated point is calculated by summing the product of the contribution probability and estimated density at each pixel, which can be reliably supervised by the ground-truth count value.

We introduce a Bayesian loss function to construct a likelihood function *p* between 2D pixel positions in the rape flower clusters image for the given rape flower clusters labels, which is defined as:

1 $$\begin{aligned} p\left( {\textbf{x}}_{m} \mid y_{n}\right) ={\mathcal {N}}\left( {\textbf{x}}_{m}; {\textbf{z}}_{n}, \sigma ^{2} {\textbf{1}}_{2 \times 2}\right) \end{aligned}$$

where $m=1,2, \ldots , M$; *M* is the number of pixels in the density map; $n=1,2, \ldots , N$, *N* is the total flower clusters count, ${\textbf{x}}_{m}$ is a 2D pixel location in the rape flower clusters image, $y_{n}$ is a given rape flower clusters labeled point location, ${\mathcal {N}}\left( {\textbf{x}}_{m}; {\textbf{z}}_{n}, \sigma ^{2} {\textbf{1}}_{2 \times 2}\right)$ is a 2D Gaussian distribution evaluated at ${\textbf{x}}_{m}$, ${\textbf{z}}_{n}$ is the mean of that labeled point, and $\sigma ^{2} {\textbf{1}}_{2 \times 2}$ is an isotropic covariance matrix.

According to Bayes’ theorem and assuming equal prior probabilities for each labeled $y_{n}$, i.e., $p\left( y_{n}\right) = \frac{1}{N}$, the equation can be simplified after introducing the posterior probabilities as,

2 $$\begin{aligned} \begin{aligned} p\left( y_{n} \mid {\textbf{x}}_{m}\right) =\frac{{\mathcal {N}}\left( {\textbf{x}}_{m}; {\textbf{z}}_{n}, \sigma ^{2} {\textbf{1}}_{2 \times 2}\right) }{\sum _{n=1}^{N} {\mathcal {N}}\left( {\textbf{x}}_{m}; {\textbf{z}}_{n}, \sigma ^{2} {\textbf{1}}_{2 \times 2}\right) } \end{aligned} \end{aligned}$$

Using the above posterior probability and density estimation map $D_{est}$, we can obtain the Bayesian loss function,

3 $$\begin{aligned} {\mathcal {L}}^{\text{ Bayes } }=\sum _{n=1}^{N} {\mathcal {F}}\left( 1-\sum _{m=1}^{M} p\left( y_{n} \mid {\textbf{x}}_{m}\right) {\textbf{D}}^{e s t}\left( {\textbf{x}}_{m}\right) \right) \end{aligned}$$

The presence of background pixel points will have a great impact on the regression estimation. In order to further eliminate the bias brought by the background pixels on the loss function, the background points outside the annotation are treated as another annotation $y_{0} = 0$. At this time the loss function can be expressed as,

4 $$\begin{aligned} \begin{aligned} {\mathcal {L}}^{\text{ Bayes } +}=&\sum _{n=1}^{N} {\mathcal {F}}(1- \sum _{m=1}^{M} p\left( y_{n} \mid {\textbf{x}}_{m}\right) {\textbf{D}}^{e s t}\left( {\textbf{x}}_{m}\right) \\&- \sum _{m=1}^{M} p\left( y_{0} \mid {\textbf{x}}_{m}\right) {\textbf{D}}^{e s t}\left( {\textbf{x}}_{m}\right) ) \end{aligned} \end{aligned}$$

To define the background likelihood, we construct a dummy background point for each pixel,

5 $$\begin{aligned} {\textbf{z}}_{0}^{m}={\textbf{z}}_{n}^{m}+d \frac{{\textbf{x}}_{m}-{\textbf{z}}_{n}^{m}}{\left\| {\textbf{x}}_{m}-{\textbf{z}}_{n}^{m}\right\| _{2}} \end{aligned}$$

where ${\textbf{z}}_{n}^{m}$ is the nearest rape flower clusters labeled centroid to ${\textbf{x}}_{m}$, ${\textbf{z}}_{0}^{m}$ is the background point, and *d* is the distance between this centroid and the background point ${\textbf{z}}_{0}^{m}$.

**Fig. 7:**
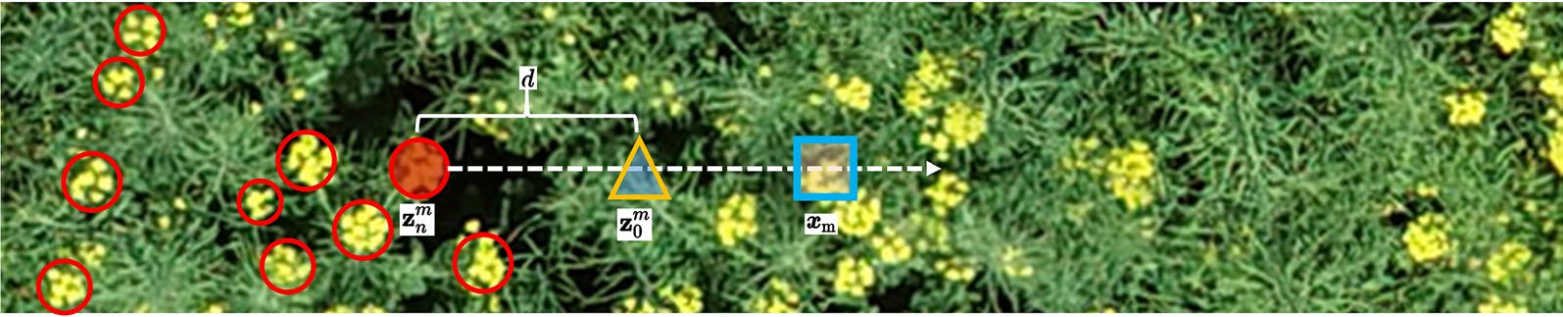
Schematic diagram of background points

To eliminate the effect of large backgrounds on regression estimation, the background information is treated as a class and its posterior probability is calculated. In the case where the defined virtual background point z_0_^m^ is 0, for pixels x^m^ away from the head point, it can be assigned to the background label, and the geometry is illustrated in Fig. 7. We also use the Gaussian kernel to define the background likelihood,

6 $$\begin{aligned} \begin{aligned} p\left( {\textbf{x}}_{m} \mid y_{0}\right)&{\mathop {=}\limits ^{ \text{ def } }} {\mathcal {N}}\left( {\textbf{x}}_{m}; {\textbf{z}}_{0}^{m}, \sigma ^{2} {\textbf{1}}_{2 \times 2}\right) \\&=\frac{1}{\sqrt{2 \pi } \sigma } \exp \left( -\frac{\left( d-\left\| {\textbf{x}}_{m}-{\textbf{z}}_{n}^{m}\right\| _{2}\right) ^{2}}{2 \sigma ^{2}}\right) \end{aligned} \end{aligned}$$

### RapeNet+

#### Coordinate attention

**Fig. 8:**
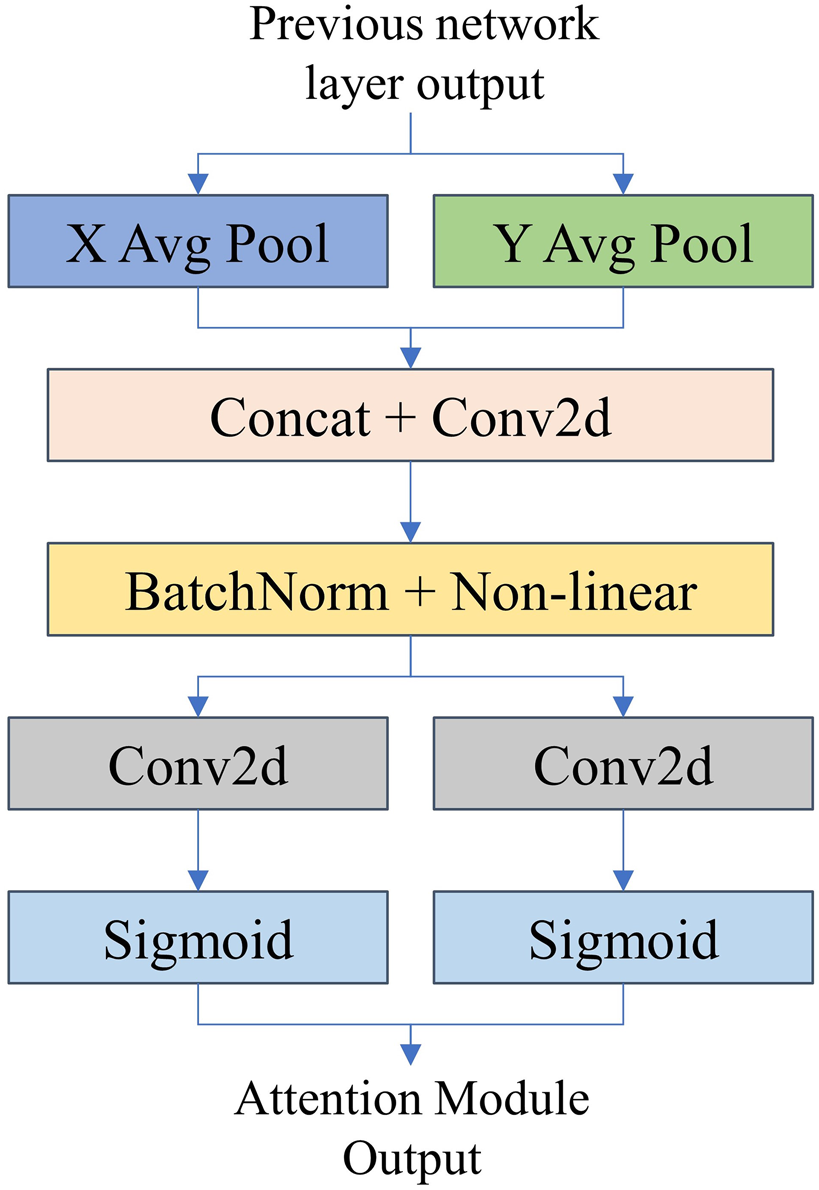
CA attention structure

Several studies have shown that introducing channel attention modules through branching strategies in the design of deep learning networks can improve the performance of models [42–45]. It enables lightweight networks to pay attention to larger areas by embedding location information into channel attention while avoiding incurring large computational overhead. Additionally, the coordinate attention module can be flexibly inserted into deep learning networks [46]. The structure of the coordinate attention module is shown in Fig. 8. ‘X Avg Pool’ and ‘Y Avg Pool’ refer to 1D horizontal global pooling and 1D vertical global pooling, respectively. The attention module uses two one-dimensional global pooling operations to aggregate the input features in the vertical and horizontal directions into two independent direction-aware feature maps. These two feature maps, embedded with direction-specific information, are then encoded as two attention maps.

**Fig. 9:**
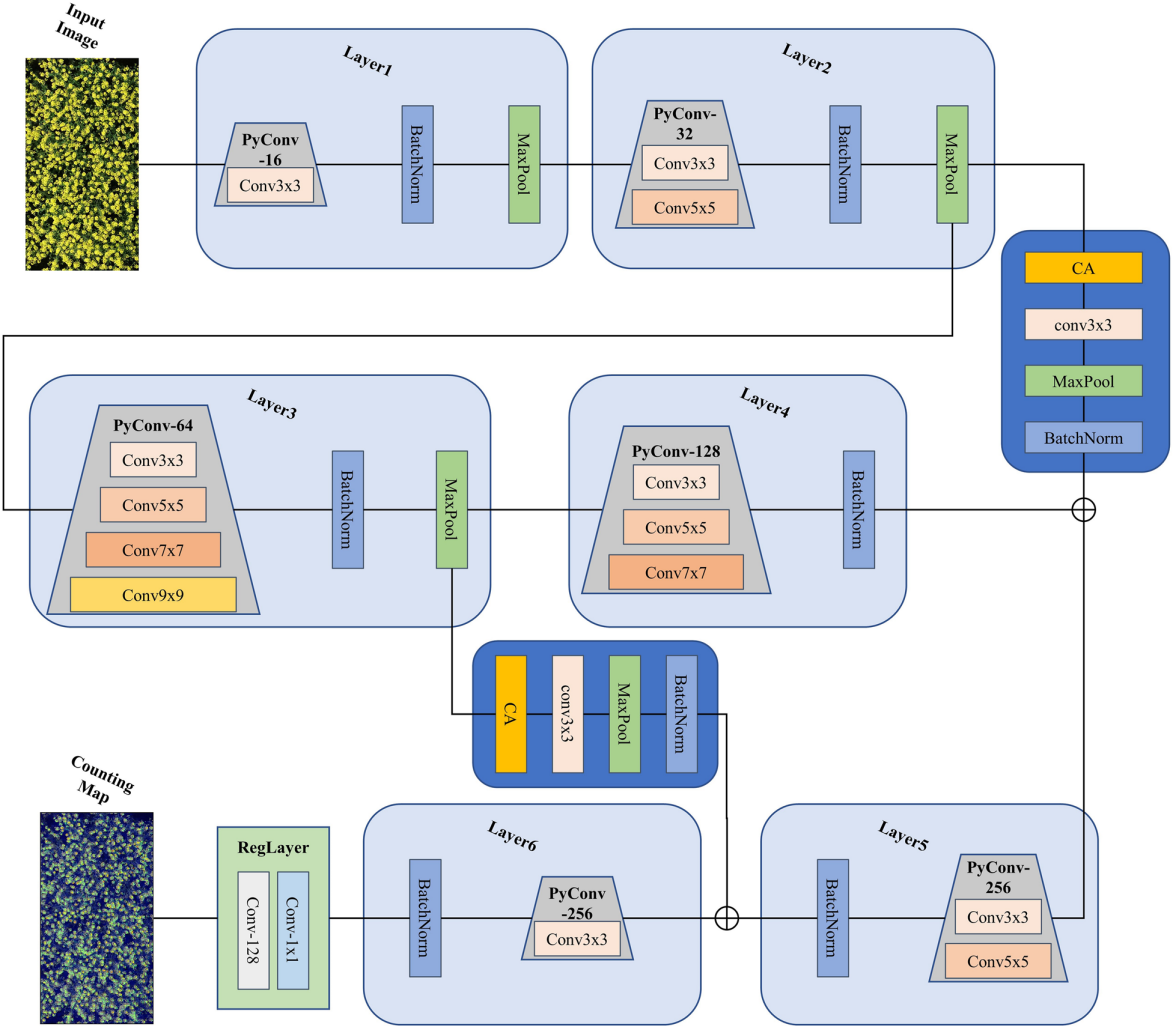
RapeNet+ network structure

The structure of the RapeNet+ network is shown in Fig. 9. A coordinate attention branching structure is introduced after the second and fourth layers of pyramidal convolution, respectively. The convolutional pooling and normalization layers are added to perform the transformation of the feature channels.

### Evaluation metrics

The performance of rape counting model is analyzed by using the common evaluation indexes of regression counting, which are Average Accuracy (Acc), Mean Absolute Error (MAE), Mean Squared Error (MSE), root Mean Absolute Error (rMAE), root Mean Squared Error (rMSE), relative root Mean Square Error (rrMSE) and $R^2$ [28, 29, 47–49]. The smaller the values of the metrics MAE, MSE, rMAE, rMSE, and rrMSE, the closer the values of the metrics Acc, and $R^2$ are to 1, which indicates the better performance of the model. The superiority of these evaluation metrics in this study indicates the high accuracy and robustness of rape flower clusters counting. The formulas for these metrics are as follows:

7 $$\begin{aligned} A c c= & {} \left( 1-\frac{1}{n} \sum _{i=1}^{n} \frac{\left| M_{i}-I_{i}\right| }{M_{i}}\right) \times 100 \% \end{aligned}$$

8 $$\begin{aligned} M A E= & {} \frac{1}{n} \sum _{i=1}^{n}\left| M_{i}-I_{i}\right| \end{aligned}$$

9 $$\begin{aligned} r M A E= & {} \sqrt{\frac{1}{n} \sum _{i=1}^{n}\left| M_{i}-I_{i}\right| } \end{aligned}$$

10 $$\begin{aligned} r M S E= & {} \sqrt{\frac{1}{n} \sum _{i=1}^{n}\left( M_{i}-{I}_{i}\right) ^{2}} \end{aligned}$$

11 $$\begin{aligned} r r M S E= & {} \frac{r M S E}{\bar{M}} \times 100 \end{aligned}$$

12 $$\begin{aligned} R^{2}= & {} 1-\frac{\sum _{i=1}^{n}\left( M_{i}-I_{i}\right) ^{2}}{\sum _{i=1}^{n}\left( I_{i}-\bar{I}\right) ^{2}} \end{aligned}$$

where *n* is the number of images, $M_{i}$ is the manual count of rape flower clusters in image *i*, $I_{i}$ is the inferred count of rape flower clusters in image *i*, $\bar{M}$ is the average manual count of rape flower clusters per image, and $\bar{I}$ is the average inferred count of rape flower clusters per image.

## Results

In this section, we describe the rape flower cluster dataset settings, training details, and experimental results used for the experiments.

### Training details

We opted for the PyTorch deep learning framework to build the network model and conducted experiments on an NVIDIA 3080 graphics card. Eighty-five percent of the data was used for the training set and the remaining $15\%$ for the test set, where the training and validation in the training set were divided 9:1. The learning rate is set to 0.00001. The SGD method is used to optimize the learning rate in the training process. We optimize parameters for 1000 epochs with a batch size of 1. The sigma value ranges from 0.1 to 10, and the background ratio ranges from 0 to 1.

### Results on RFRB

#### Regression counting network performance validation

To do research on the counting method of rape flower clusters, we conducted the experiment on rectangular box labeled RFRB dataset. In all the following regression networks, we converted the rectangular box coordinate information into centroid coordinates for our experiments. To verify the performance of bayesian losses on the regression counting network, we used different losses on the rape flower clusters dataset RFRB for comparison experiments. The classical Vgg19 and RapeNet+ networks are chosen as the backbone networks for the loss function comparison experiments.

Among the loss functions used for comparison, OT loss [34] and TV loss [34] are training constraints on the differences between distributions of normalized density functions. The MAE loss and MSE loss [50, 51] are trained to constrain the difference values and the sum of squares of the difference values between the predicted and manually labeled, respectively. DM loss [34] integrates the values of TV, OT, MAE for training constraints. From the comparison results shown in Table 3, we can see that the performance of MAE loss, MSE loss, DM loss and Bayesian loss is better than OT loss or TV loss alone. Among them, Bayesian loss has the highest Acc and $R^2$ in the RFRB dateset, up to 0.9098 and 0.9623, respectively. For the measures of MAE, rMAE, rMSE, and rRMSE, the bayesian loss has a lower value compared with other loss methods. The better accuracy and less counting error illustrate the effectiveness of density contribution probability model constrained by bayesian loss.

**Table 3:** Comparison of different losses on RFRB

Backbone	Loss	Acc	MAE	rMAE	rMSE	rrMSE	$R^2$
Vgg19	OT	0.6499	108.07	10.40	128.08	38.77	0.6492
	TV	0.7132	82.91	9.12	97.61	30.16	0.2587
	MAE	0.8981	25.94	5.09	34.43	12.56	0.9512
	MSE	0.9027	24.40	4.94	32.91	11.70	0.9534
	DM	0.9014	24.43	4.94	32.38	12.99	0.9589
	Bayesian	0.9098	22.90	4.78	31.95	12.15	0.9623
RapeNet+	OT	0.8306	45.31	6.73	60.69	19.75	0.8841
	TV	0.4787	149.05	12.21	168.70	52.71	–
	MAE	0.8733	37.06	6.09	49.67	14.32	0.8658
	MSE	0.8896	29.96	5.47	42.27	13.47	0.9428
	DM	0.9017	23.10	4.81	32.08	12.28	0.9613
	Bayesian	0.9026	23.65	4.86	29.95	12.03	0.9635

To analyze the performance of the proposed network, we compared its counting performance with some classical backbones combined with Bayesian loss. For each of these popular backbones, there are several networks with different hierarchical structures from which to choose. To ensure that the training results do not lose generality, we select network structures with the same magnitude of model capacity for comparison experiments, namely Mnasnet0_75 [52], Densenet121 [53], Efficientnet_b3 [54], and Vgg19 [37]. These backbones are generally designed to perform tasks such as classification and segmentation. In the regression counting task of this paper, we need to change the tail structure of these backbone networks to output the counting results we want. We replace the classification layer at the tail of these backbone networks with a regression layer that is consistent with the structure of the RapeNet proposed in this paper. The number of channels is adjusted according to the output of the previous network layer to better articulate the regression layer. For consistency, the data set allocation ratio was set to be the same, and 256$\times$256 was used as input in all regression estimation networks.

The experimental results are shown in Table 4. We introduced the model capacity in addition to the basic evaluation metrics to further evaluate the model. It can be seen that the RapeNet series network model performs well on all evaluation metrics. Note that RapeNet+ has the lowest value of *rrMSE* 12.03 and the highest value of $R^2$ 0.9635. In particular, the capacity of the proposed RapeNet series is reduced by an order of magnitude, from 58.7MB to 5.8MB. In cases where the accuracy is the same, we use fewer parameters and a simpler network to complete the counting work.

**Fig. 10:**
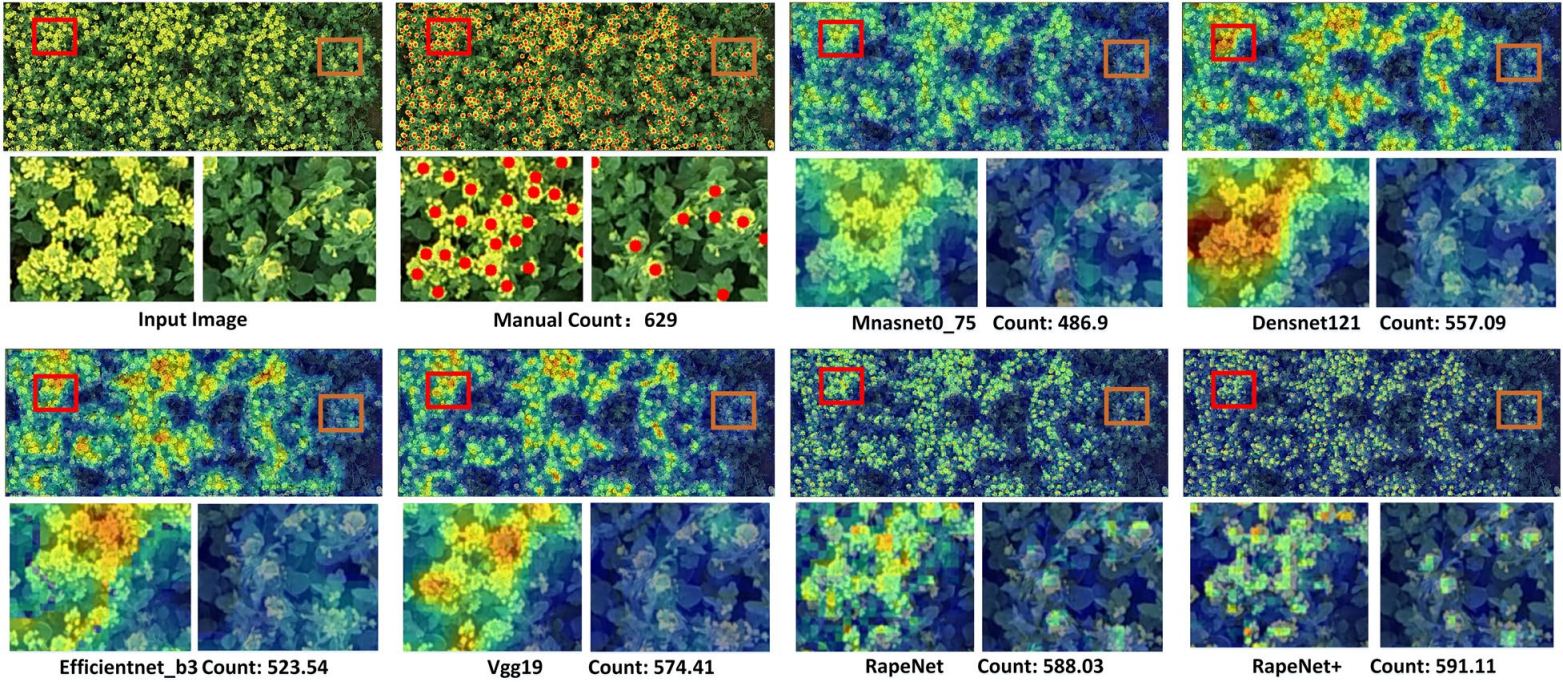
Visual results of different backbones on RFRB

Figure 10 shows the visual comparative result of the heat maps under six different skeletons. The enlarged subimages, including images of a dense part and a sparse part, come from the heat maps with the same location. It can be seen that the proposed methods pay more attention to flowers compared with other methods. The RapeNet series distinguishes most of the overlapped rape flower clusters. Besides, it is clear which one is counted and which is not, providing a better explanation of the heat map. As a consequence, the counting results of the RapeNet series are closer to a manual count. The RapeNet series is suitable for large-scale and high-throughput counting of rape flower clusters.

**Table 4:** Comparison of different backbones on RFRB

Methods		Acc	MAE	rMAE	rMSE	rrMSE	R$^2$	Model capacity
Backbone	Loss							
$Mnasnet0\_75$	Bayesian loss	0.7100	53.99	7.35	65.08	47.90	0.8278	20.8MB
*Densenet*121		0.8679	27.38	5.23	32.58	17.13	0.9568	53.2MB
$Efficientnet\_b3$		0.9047	28.34	5.32	38.89	11.61	0.9385	58.7MB
*Vgg*19		0.9098	22.90	4.78	31.95	12.15	0.9623	86.0MB
*RapeNet*		0.8981	25.31	5.03	32.73	13.21	0.9566	4.9MB
$RapeNet+$		0.9062	23.65	4.86	29.95	12.03	0.9635	5.6MB

#### Comparison of detection counting and regression counting methods

There are two different types of solutions for the counting of rape flower clusters. One is an object detection technique that involves counting the number of bounding boxes. Another promising paradigm is termed “object counting,” where plant counts are regressed directly from images without detecting bounding boxes [30]. To verify the counting performance of these two different types of methods on rape flower clusters, we performed experimental validation on the rectangular box-labeled dataset RFRB.

Three classical detection networks, Faster-Rcnn, YOLOv4, and Centernet, were used to count rape flower clusters in the form of a detection bounding box. Faster-Rcnn, as a typical two-stage target detection algorithm, discards the traditional sliding window and SS (Selective Search) methods. It chooses RPN to generate the detection box, which greatly improves the detection box generation speed. YOLOv4 combines the recent optimization strategies in the CNN field and optimizes the data processing, backbone network, activation function, loss function, etc. to achieve a good balance between detection accuracy and running speed. Through several experiments, the bounding box confidence lower limit was set to 0.5 to get the most suitable counting box. The Centernet network, as an excellent member of the anchor-free model, has the feature of a large output resolution with only four downsampling rates and a good detection effect for small targets. Similarly, the confidence lower limit value is set to 0.6 to obtain the counting frame after several trials. Two regression counting networks, TasselNetV2+ and DM-Count, were counted as centroids. The four vertex coordinate values in the top, bottom, left, and right of the manual labeling frame are converted into one centroid coordinate value. Then, the centroid coordinate values are used as labels for the counting study.

**Fig. 11:**
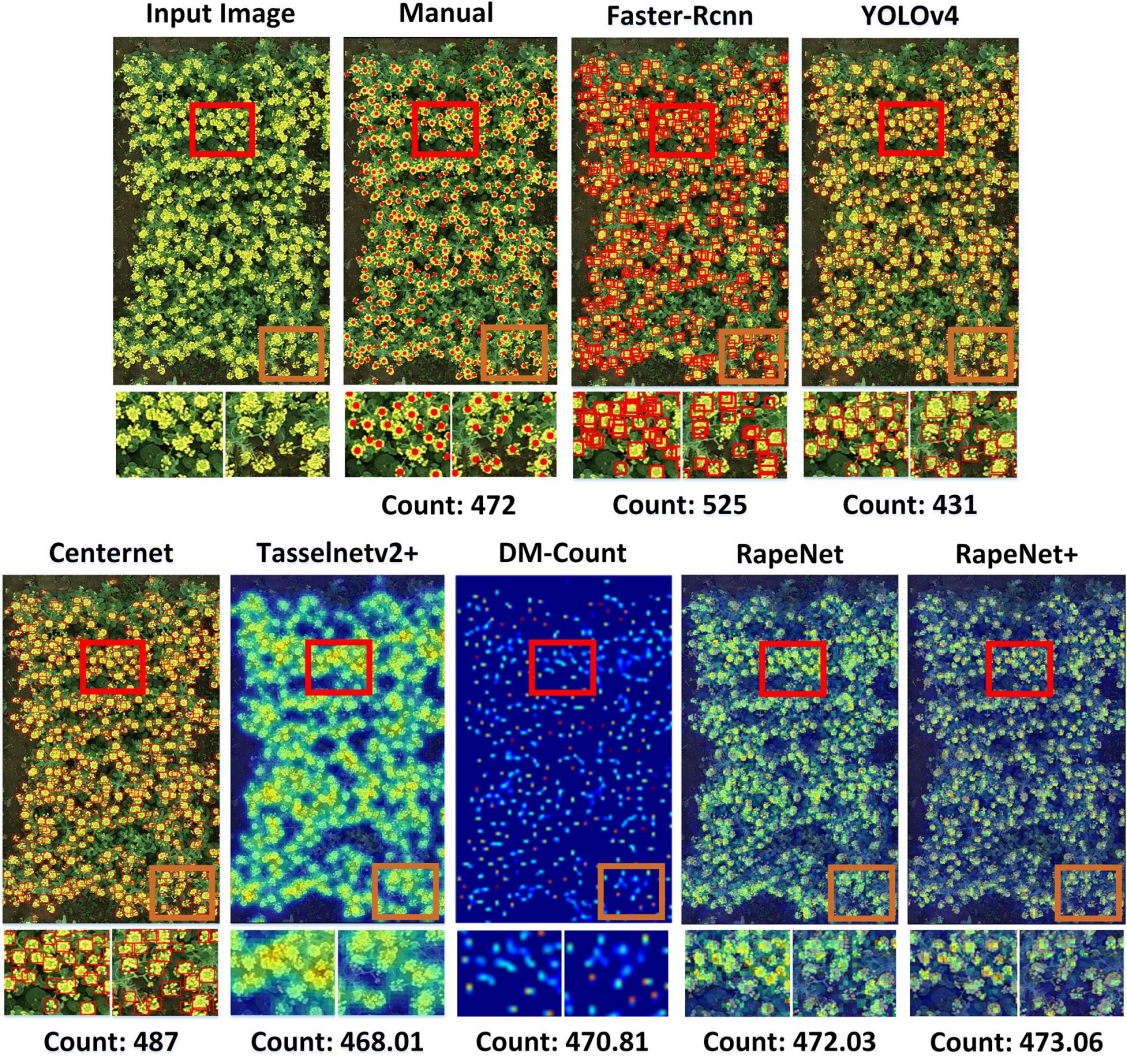
RFRB experiment results visualization

An input test image, a corresponding manually labeled image, and the effect plots and counting results of the outputs of six different methods are shown in Fig. 11. The results of three detection networks, Faster-Rcnn, YOLOv4, and Centernet, are shown in the form of detection boxes, and the results of two regression estimation networks, TasselNetV2+ and DM-Count, are shown in the form of heat plots. From the subimages in Fig. 11, we can see that there are more duplicate bounding boxes in the Faster-Rcnn detection results. It indicates that the network has poor detection and serious duplicate in dense flower area. We observed that YOLOv4 has more missed detection since the inability of the detection boxes to distinguish the edges of rape flower clusters. YOLOv4 is not sensitive to the detection of small and dense rape flower clusters in UAV images, especially for the more dense and heavily adhered obscured areas. This detection result is similar in UAV image target detection [55]. The Centernet network has a better detection effect, with fewer false detections and omissions, and the final count result value is closer with the manual count value. TasselNetV2+ and DM-Count perform comparably from the Fig. 11, and the count results do not deviate much from the manual count values.

Extended comparative experiments are shown in Table 5, which is about the performance of various counting methods on different assessment metrics in the test images of RFRB. We can see the Acc is above 0.87 except for Faster−Rcnn. RapeNet series, TasselnetV2+, DM-Count networks outperformed Centernet, YOLOv4 and Faster-Rcnn networks in terms of the values of each metric. Small and dense objects are prone to missed detection or error detection when using object detection. The indicators show the good applicability of the regression counting method in rape flower cluster counting. These are consistent with the conclusions of the visually observed results.

**Table 5:** Comparison of different networks on RFRB

Methods	Acc	MAE	rMAE	rMSE	rrMSE	$R^2$
*Faster*-*Rcnn*	0.6562	58.96	7.68	76.79	55.90	0.5738
*YOLOv*4	0.8700	35.15	5.92	50.83	17.96	0.8491
*Centernet*	0.8958	26.23	5.12	33.61	13.69	0.9472
$TasselNetV2+$	0.8984	26.58	5.16	34.72	13.09	0.9528
*DM*-*Count*	0.9014	24.43	4.94	32.38	12.99	0.9589
*RapeNet*	0.8981	25.31	5.03	32.73	13.21	0.9566
$RapeNet+$	0.9062	23.65	4.86	29.95	12.03	0.9635

### Results on RFCP

To further investigate the reliability of the counting, we conducted experimental validation on the RFCP dataset with the centroid labeled. It contains more dense rape flower clusters. Table 6 shows the results of each metric for DM-Count, TasselNetV2+, and the two regression networks proposed in this paper, RapeNet and RapeNet+. Experimental results show that all four networks performed well on the RFCP dataset. The Acc metrics of the TasselNetV2+, RapeNet, and RapeNet+ networks are comparable, and the DM-Count network is slightly lower. The best values were obtained for MAE, rMAE, rMSE, rrMSE, and R2 metrics by the RapeNet+ network.

**Table 6:** Comparison of different networks on RFCP

Methods	Acc	MAE	rMAE	rMSE	rrMSE	$R^2$
*DM*-*Count* [34]	0.9464	30.53	5.53	45.16	7.04	0.9460
$TasselNetV2+$	0.9502	22.77	4.77	32.91	5.66	0.9735
*RapeNet*	0.9544	25.87	5.09	33.21	5.39	0.9701
$RapeNet+$	0.9573	22.96	4.79	27.41	4.82	0.9870

In order to verify the applicability of the proposed method in different resolution, we adjusted the input resolution of each group of networks by multiplying the original images by coefficients of 0.8 and 0.5, respectively. Table 7 shows the results of different resolutions on RFCP for each metric for DM-Count, TasselNetV2+, and the two regression networks, RapeNet and RapeNet+. The proposed networks and TasselNetV2+ counting proposed in this paper have comparable performance at the original image resolution. At a factor of 0.8, the performance has little change in indicators. When the factor is 0.5, the counting performance of the DM-Count and TasselNetV2+ networks degrade sharply. However, our proposed RapeNet and RapeNet+ networks maintain the performance at coefficients of 0.8 and 0.5, which is also due to the fact that our backbone is built from multilayer pyramidal convolution and can adapt to UAV rape cluster counting at multi-scale resolution.

**Table 7:** Comparison of different resolutions on RFCP

Methods	Resize	Acc	MAE	rMAE	rMSE	rrMSE	$R^2$
*DM*-*Count*	1.0	0.9464	30.53	5.53	45.16	7.04	0.9460
	0.8	0.9417	27.17	5.21	32.20	7.25	0.9787
	0.5	0.8747	60.32	7.77	64.65	14.03	0.9074
$TasselNetV2+$	1.0	0.9502	22.77	4.77	32.91	5.66	0.9735
	0.8	0.9558	22.99	4.80	31.55	5.69	0.9756
	0.5	0.3282	356.56	18.88	373.30	67.47	−
*RapeNet*	1.0	0.9544	25.87	5.09	33.21	5.39	0.9701
	0.8	0.9543	25.87	5.09	33.21	5.39	0.9701
	0.5	0.9543	25.87	5.09	33.21	5.39	0.9701
$RapeNet+$	1.0	0.9573	22.96	4.79	27.41	4.82	0.9870
	0.8	0.9573	22.96	4.79	27.41	4.82	0.9870
	0.5	0.9573	22.96	4.79	27.41	4.82	0.9870

## Discussion

Developing low-cost, fast, and field-based counting methods to assess the number of flower clusters can improve the study of rapeseed phenotype and help establish a more comprehensive yield prediction model. The UAV path planning and control system can be used to easily obtain standardized and uniform high-resolution RGB aerial images of large fields. Then, the flower cluster statistics of the corresponding field are obtained by analyzing the RGB images through the deep learning method. We collected datasets RFRB and RFCP containing 24 classes of rapeseed material in fields between 2021 and 2022, including 51,136 manually annotated rectangular boxes on the RFRB dataset and 104,391 manually annotated points on the RFCP dataset.

To count the number of rape flower clusters in a large field environment, we conducted an exploratory study of various counting methods, such as the target detection-based counting method. Target detection is trained by deep learning directly between the manually annotated boxes and the corresponding images to derive a prediction model. Excellent detection networks such as YOLOv4, Faster-Rcnn, and Centernet work well in terms of evaluating metrics (Table 5). Centernet, as a traditional anchor-free model, is better at detecting flower clusters. However, labeling manually annotated rectangular boxes is difficult in dense clusters.

In the following experiment, we investigate a counting method based on regression estimation. The center point annotation is relatively simpler and faster to operate. Tasselnetv2+ performs well on crop counts such as wheat ears and corn stamens. This deep learning network has outstanding results on RFRB, with $R^2$ up to 0.95. Additionally, we use the DM-count in our experiment, which is a classical method in crowd counting. The highly correlated results with manual counting demonstrate($R^2=0.95$) that it is reliable to use the regression estimation method to complete the counting task of rape flower clusters. Consequently, we designed RapeNet and RapeNet+ to count rapeseed flower clusters.

**Fig. 12:**
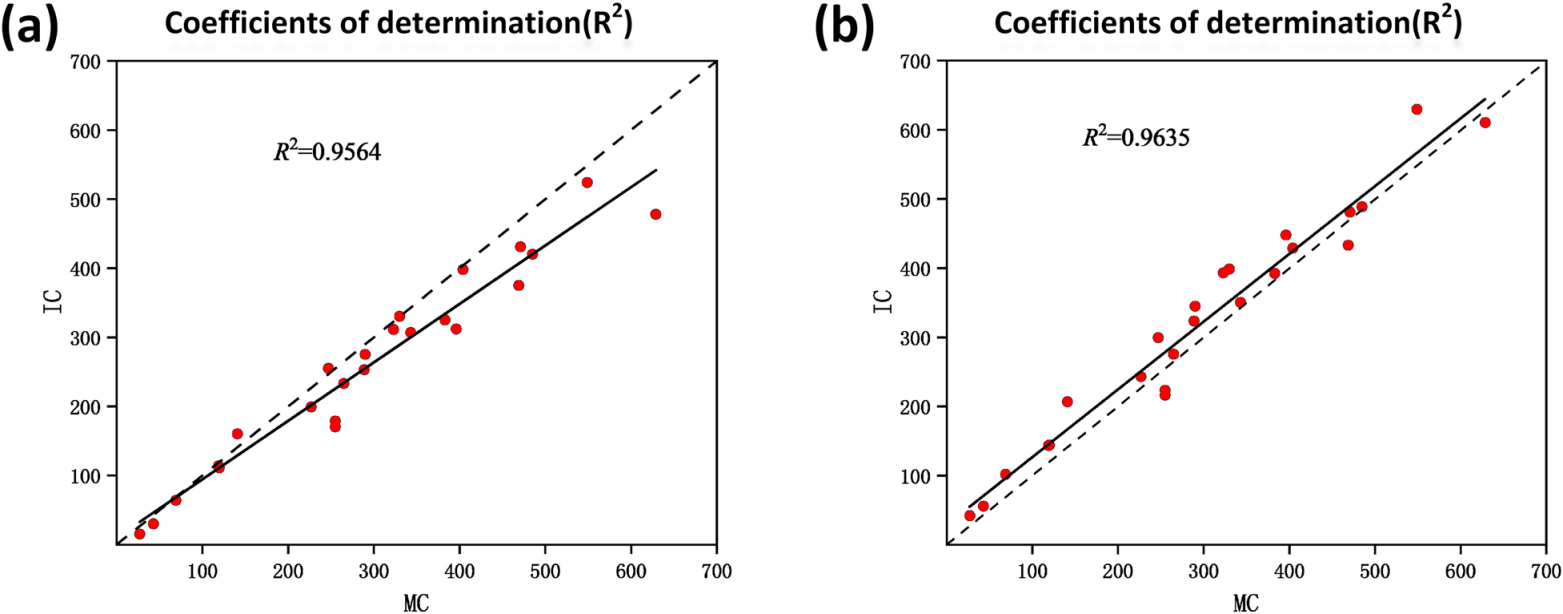
The coefficients of determination of **a** RapeNet and **b** RapeNet+ on RFRB

The coefficient of determination ($R^2$) was exploited to reflect the fitting degree of the linear regression model, representing the interpretation degree of the total number of rape flower clusters in each plot to the seed yield. The count number of the rape flower clusters predicted by the proposed network model for each plot and the corresponding manual number were recorded to explore the correlation between them. We show the results of the RapeNet network and the RapeNet+ network in Fig. 12a and b. A strong correlation between manual count (MC) and inferred count (IC) is observed on the RFRB dataset, with an $R^2$ of 0.9564 and 0.9635, respectively. This demonstrates that most of the predictions are sufficiently accurate. Compared with RapeNet, the fitted curve of RapeNet+ is closer to 1:1. This is because the corresponding model learned by RapeNet+ with the attention mechanism on this training set may generalize well to the testing set with significant variations in plant cultivars, illumination changes, and poses. On the whole, the RapeNet and RapeNet+ networks proposed in this paper perform well and can be used in the rape flower cluster counting.

**Fig. 13:**
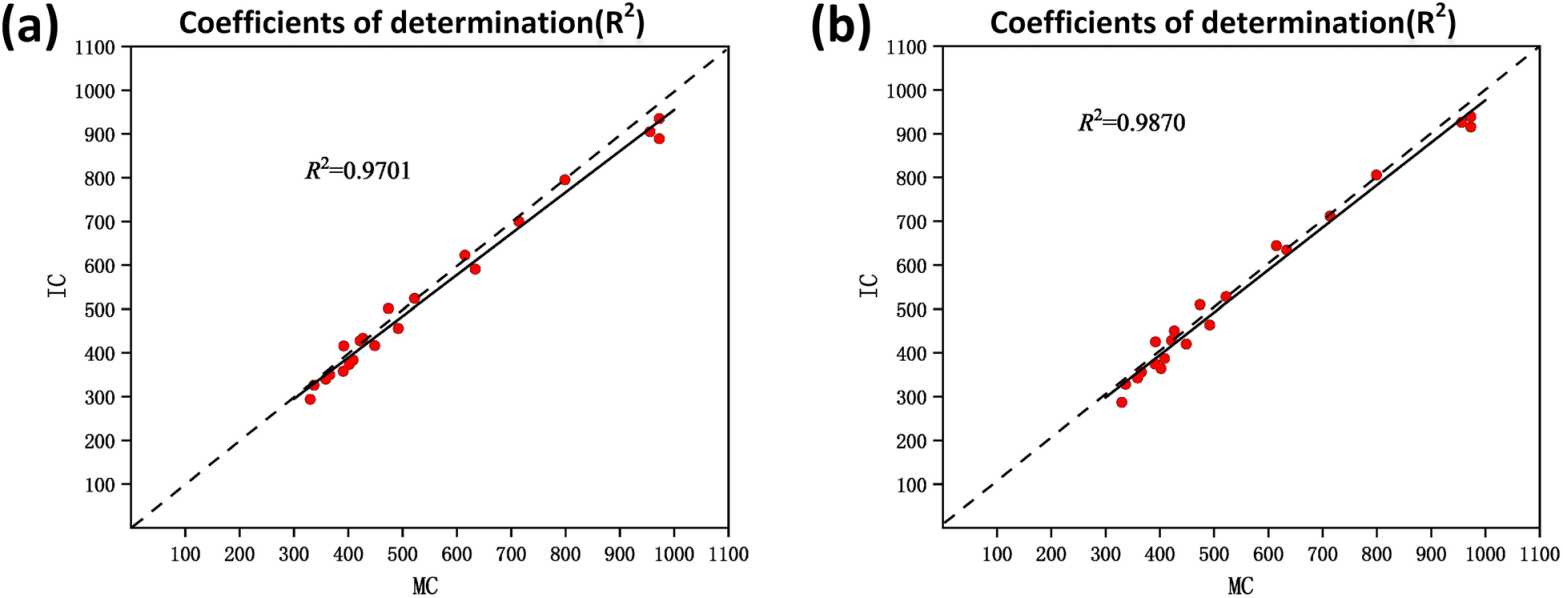
The coefficients of determination of **a** RapeNet and **b** RapeNet+ on RFCP

The peak period of rape flowers has strong agronomic significance [11]. But Dense and adherent flowers make counting difficult. The counting performance of the DM-count method has decreased in RFCP as expected. TasselNetv2+ and the proposed RapeNet series improved a little in this case. Especially, the value of $R^2$ in RapeNet+ is 0.98. Figure 13a and b further show the correlation between the RapeNet network and the RapeNet+ network in terms of manual counting and inferred counting. It can be observed that a lot of the counts are accurate. However, as the number of flowers increases, there are more counting errors, which shows that dense counting is indeed a difficult task.

**Fig. 14:**
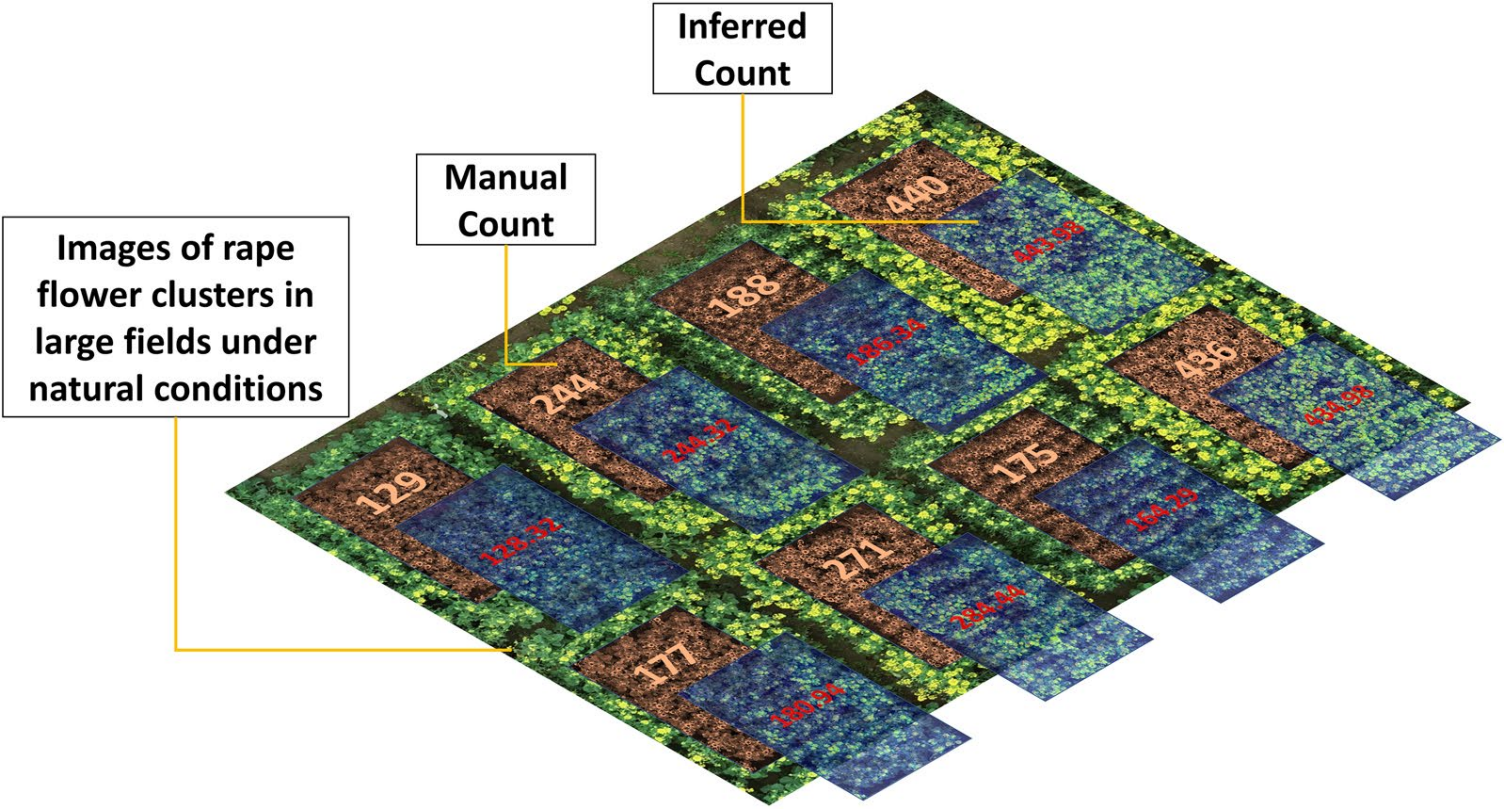
Images of rape flower clusters and prediction of rape flower clusters number in a field under natural conditions

Figure 14 shows the performance of counting rape flower clusters under natural conditions in a large field scenario captured in 2022. The result illustrates the good counts for different types of rape flower clusters in different field areas. Flower clusters are quantified, which provides a good theoretical basis for agronomists and breeders to study the relationship between rape phenotype and yield.

## Conclusions

Application of modern technologies to the management of rapeseed will greatly increase the harvest. It is also helpful for breeders to analyze the phenotypic traits of the material and breed for improved yield. In this paper, we discuss and validate the rape flower cluster counting method on two rape flower cluster labeling datasets, Rape Flower Rectangular Box Labelling (RFRB) and Rape Flower Center Point Labelling (RFCP). The images used in the dataset were acquired remotely from the UAV with an RGB camera in a field. We proposed a RapeNet series networks using pyramidal convolution. RapeNet is a lightweight deep regression network that performs regression counting of rape clusters and combines it with a Bayesian loss function for constraint. The proposed networks incorporate the advantages of mainstream regression estimation methods, which rely on powerful feature extraction capabilities. These improve the robustness of high-throughput counting in high-resolution images. Because overlap and adhesion are more severe in densely clustered images of rape flower clusters. To further improve the accuracy of rape flower cluster counting, we extended the RapeNet to a network with a branch attention mechanism, RapeNet+. Experiments show the proposed method can predict the number of rape flower clusters in a UAV image accurately. It also improves the applicability of deep learning-based counting networks in rape flower clusters with low-cost labelling. The comprehensive analysis of the experimental results shows that the proposed methods count rape flower clusters in large field scenarios with high efficiency, which can better meet the requirements of practical applications and provide a new method for rape flower cluster counting. In our future work, we will continue to study the counting and the coverage of rape flower clusters in large field scenarios. We also tend to make full use of the complementarity of detection and regression counting models to investigate the cross-domain method based on detection-regression bidirectional knowledge migration to further improve counting performance. Additionally, the relationship between flowing data and the yield prediction model will be considered.

## Data Availability

The codes, datasets RFRB and RFCP used in the study are available online at: https://github.com/CV-Wang/RapeNet.

## References

[1] Zhang X, He Y (2013). Rapid estimation of seed yield using hyperspectral images of oilseed rape leaves. Ind Crops Prod.

[2] Amiri M, Raeisi-Dehkordi H, Sarrafzadegan N, Forbes SC, Salehi-Abargouei A (2020). The effects of canola oil on cardiovascular risk factors: a systematic review and meta-analysis with dose–response analysis of controlled clinical trials. Nutr Metab Cardiovasc Dis.

[3] George B, Loeser E (2021). Oilseeds: world markets and trade.

[4] Asare E, Scarisbrick D (1995). Rate of nitrogen and sulphur fertilizers on yield, yield components and seed quality of oilseed rape (brassica napus l.). Field Crops Res.

[5] Luo Y (2022). On farm harvest and storage losses of oil crops and the impact on resources and environment in China. Chin J Oil Crop Sci.

[6] Stankevych S, Yevtushenko M, Vilna V, Zabrodina I, Yushchuk D, Sirous LY, Lutytska N, Molchanova O, Melenti V, Golovan L (2019). Efficiency of chemical protection of spring rape and mustard from rape blossom beetle. Ukrain J Ecol.

[7] Riar A, Gill G, McDonald G (2020). Different post-sowing nitrogen management approaches required to improve nitrogen and water use efficiency of canola and mustard. Front Plant Sci..

[8] Bouchet A-S, Laperche A, Bissuel-Belaygue C, Snowdon R, Nesi N, Stahl A (2016). Nitrogen use efficiency in rapeseed. A review. Agron Sustain Dev.

[9] Diepenbrock W (2000). Yield analysis of winter oilseed rape (brassica napus l.): a review. Field Crops Res.

[10] Behrens T, Müller J, Diepenbrock W (2006). Utilization of canopy reflectance to predict properties of oilseed rape (brassica napus l.) and barley (hordeum vulgare l.) during ontogenesis. Eur J Agron.

[11] d’Andrimont R, Taymans M, Lemoine G, Ceglar A, Yordanov M, van der Velde M (2020). Detecting flowering phenology in oil seed rape parcels with sentinel-1 and -2 time series. Rem Sens Environ.

[12] Feng A, Zhou J, Vories E, Sudduth KA (2020). Evaluation of cotton emergence using uav-based imagery and deep learning. Comput Electron Agric.

[13] Oh S, Chang A, Ashapure A, Jung J, Dube N, Maeda M, Gonzalez D, Landivar J (2020). Plant counting of cotton from uas imagery using deep learning-based object detection framework. Rem Sens.

[14] Wang L, Xiang L, Tang L, Jiang H (2021). A convolutional neural network-based method for corn stand counting in the field. Sensors.

[15] Li L, Zhang Q, Huang D (2014). A review of imaging techniques for plant phenotyping. Sensors.

[16] Vikram P, Anand N, Linesh R (2017). Agriculture drones: a modern breakthrough in precision agriculture. Int J Rem Sens.

[17] Feng L, Chen S, Zhang C, Zhang Y, He Y (2021). A comprehensive review on recent applications of unmanned aerial vehicle remote sensing with various sensors for high-throughput plant phenotyping. Comput Electron Agric.

[18] Fang S, Tang W, Peng Y, Gong Y, Dai C, Chai R, Liu K (2016). Remote estimation of vegetation fraction and flower fraction in oilseed rape with unmanned aerial vehicle data. Rem Sens..

[19] Wan L, Li Y, Cen H, Zhu J, Yin W, Wu W, Zhu H, Sun D, Zhou W, He Y (2018). Combining uav-based vegetation indices and image classification to estimate flower number in oilseed rape. Rem Sens..

[20] Zang Y, Chen X, Chen J, Tian Y, Shi Y, Cao X, Cui X (2020). Remote sensing index for mapping canola flowers using modis data. Rem Sens..

[21] Zhang T, Vail S, Duddu HSN, Parkin IAP, Guo X, Johnson EN, Shirtliffe SJ (2021). Phenotyping flowering in canola (brassica napus l.) and estimating seed yield using an unmanned aerial vehicle-based imagery. Front Plant Sci..

[22] Sulik JJ, Long DS (2015). Spectral indices for yellow canola flowers. Int J Rem Sens.

[23] Zhang G, Zhao S, Li W, Du Q, Ran Q, Tao R (2020). Htd-net: a deep convolutional neural network for target detection in hyperspectral imagery. Rem Sens.

[24] Gouiaa R, Akhloufi MA, Shahbazi M (2021). Advances in convolution neural networks based crowd counting and density estimation. Big Data Cogn Comput.

[25] Samiei S, Rasti P, Ly Vu J, Buitink J, Rousseau D (2020). Deep learning-based detection of seedling development. Plant Methods..

[26] Jiang Y, Li C, Paterson AH, Robertson JS (2019). Deepseedling: deep convolutional network and Kalman filter for plant seedling detection and counting in the field. Plant Methods.

[27] Yang B, Gao Z, Gao Y, Zhu Y (2021). Rapid detection and counting of wheat ears in the field using yolov4 with attention module. Agronomy.

[28] Lu H, Cao Z, Xiao Y, Zhuang B, Shen C (2017). Tasselnet: counting maize tassels in the wild via local counts regression network. Plant Methods.

[29] Xiong H, Cao Z, Lu H, Madec S, Liu L, Shen C (2019). Tasselnetv2: in-field counting of wheat spikes with context-augmented local regression networks. Plant Methods.

[30] Lu H, Cao Z (2020). Tasselnetv2+: a fast implementation for high-throughput plant counting from high-resolution rgb imagery. Front Plant Sci.

[31] Lu H, Liu L, Li Y-N, Zhao X-M, Wang X-Q, Cao Z-G (2021). Tasselnetv3: explainable plant counting with guided upsampling and background suppression. IEEE Trans Geosci Rem Sens.

[32] Madec S, Jin X, Lu H, De Solan B, Liu S, Duyme F, Heritier E, Baret F (2019). Ear density estimation from high resolution rgb imagery using deep learning technique. Agric For Meteorol.

[33] Liu L, Lu H, Li Y, Cao Z (2020). High-throughput rice density estimation from transplantation to tillering stages using deep networks. Plant Phenom (Washington, D.C.).

[34] Wang B, Liu H, Samaras D, Nguyen MH (2020). Distribution matching for crowd counting. Adv Neural Inf Process Syst.

[35] LeCun Y, Bottou L, Bengio Y, Haffner P (1998). Gradient-based learning applied to document recognition. Proc IEEE.

[36] Krizhevsky A, Sutskever I, Hinton GE (2017). Imagenet classification with deep convolutional neural networks. Commun ACM.

[37] Simonyan K, Zisserman A. Very deep convolutional networks for large-scale image recognition. 2014, arXiv preprint arXiv:1409.1556.

[38] Szegedy C, Liu W, Jia Y, Sermanet P, Reed S, Anguelov D, Erhan D, Vanhoucke V, Rabinovich A. Going deeper with convolutions. In: Proceedings of the IEEE conference on computer vision and pattern recognition, 2015:1–9.

[39] Targ S, Almeida D, Lyman K. Resnet in resnet: generalizing residual architectures. 2016, arXiv preprint arXiv:1603.08029.

[40] Duta IC, Liu L, Zhu F, Shao L. Pyramidal convolution: rethinking convolutional neural networks for visual recognition. (2020), arXiv preprint arXiv:2006.11538.

[41] Ma Z, Wei X, Hong X, Gong Y. Bayesian loss for crowd count estimation with point supervision. In: Proceedings of the IEEE/CVF international conference on computer vision, 2019:6142–6151.

[42] Zhu L, Geng X, Li Z, Liu C (2021). Improving yolov5 with attention mechanism for detecting boulders from planetary images. Rem Sens..

[43] Li R, Wu Y (2022). Improved yolo v5 wheat ear detection algorithm based on attention mechanism. Electronics.

[44] Dong Y, Liu Y, Kang H, Li C, Liu P, Liu Z (2022). Lightweight and efficient neural network with spsa attention for wheat ear detection. PeerJ Comput Sci.

[45] Wang Y, Qin Y, Cui J (2021). Occlusion robust wheat ear counting algorithm based on deep learning. Front Plant Sci.

[46] Hou Q, Zhou D, Feng J. Coordinate attention for efficient mobile network design. In: Proceedings of the IEEE/CVF conference on computer vision and pattern recognition, 2021:13713–13722.

[47] Wang D, Zhang D, Yang G, Xu B, Luo Y, Yang X (2021). Ssrnet: in-field counting wheat ears using multi-stage convolutional neural network. IEEE Trans Geosci Rem Sens.

[48] Ghorbani MA, Shamshirband S, Haghi DZ, Azani A, Bonakdari H, Ebtehaj I (2017). Application of firefly algorithm-based support vector machines for prediction of field capacity and permanent wilting point. Soil Tillage Res.

[49] Li M-F, Tang X-P, Wu W, Liu H-B (2013). General models for estimating daily global solar radiation for different solar radiation zones in mainland china. Energy Convers Manag.

[50] Alkhudaydi T et al. Counting spikelets from infield wheat crop images using fully convolutional networks. Neural Comput Appl. 2022:1–22.

[51] Banerjee BP, Sharma V, Spangenberg G, Kant S (2021). Machine learning regression analysis for estimation of crop emergence using multispectral uav imagery. Rem Sens..

[52] Tan M, Chen B, Pang R, Vasudevan V, Sandler M, Howard A, Le QV. Mnasnet: Platform-aware neural architecture search for mobile. In: Proceedings of the IEEE/CVF Conference on Computer Vision and Pattern Recognition, 2019:2820–2828.

[53] Iandola F, Moskewicz M, Karayev S, Girshick R, Darrell T, Keutzer K. Densenet: Implementing efficient convnet descriptor pyramids. 2014. arXiv preprint arXiv:1404.1869.

[54] Tan M, Le Q. Efficientnet: Rethinking model scaling for convolutional neural networks. In: International conference on machine learning, 2019:6105–6114. PMLR.

[55] Tan L, Lv X, Lian X, Wang G (2021). Yolov4\_drone: Uav image target detection based on an improved yolov4 algorithm. Comput Electr Eng.

